# Expanded Basal Compartment and Disrupted Barrier in Vocal Fold Epithelium Infected with Mouse Papillomavirus MmuPV1

**DOI:** 10.3390/v14051059

**Published:** 2022-05-16

**Authors:** Renee E. King, Ella T. Ward-Shaw, Rong Hu, Paul F. Lambert, Susan L. Thibeault

**Affiliations:** 1 McArdle Laboratory for Cancer Research, Department of Oncology, University of Wisconsin-Madison, Madison, WI 53705, USA; renee.king@wisc.edu (R.E.K.); etward@wisc.edu (E.T.W.-S.); plambert@wisc.edu (P.F.L.); 2 Department of Surgery, University of Wisconsin-Madison, Madison, WI 53705, USA; 3 Department of Pathology and Laboratory Medicine, University of Wisconsin-Madison, Madison, WI 53705, USA; rhu6@wisc.edu

**Keywords:** mouse, papillomavirus, larynx, vocal folds, epithelium, recurrent respiratory papillomatosis, RRP, MmuPV1

## Abstract

Laryngeal infection with low-risk human papillomaviruses can cause recurrent respiratory papillomatosis (RRP), a disease with severe effects on vocal fold epithelium resulting in impaired voice function and communication. RRP research has been stymied by limited preclinical models. We recently reported a murine model of laryngeal MmuPV1 infection and disease in immunodeficient mice. In the current study, we compare quantitative and qualitative measures of epithelial proliferation, apoptosis, differentiation, and barrier between mice with MmuPV1-induced disease of the larynx and surrounding tissues and equal numbers of uninfected controls. Findings supported our hypothesis that laryngeal MmuPV1 infection recapitulates many features of RRP. Like RRP, MmuPV1 increased proliferation in infected vocal fold epithelium, expanded the basal compartment of cells, decreased differentiated cells, and altered cell–cell junctions and basement membrane. Effects of MmuPV1 on apoptosis were equivocal, as with RRP. Barrier markers resembled human neoplastic disease in severe MmuPV1-induced disease. We conclude that MmuPV1 infection of the mouse larynx provides a useful, if imperfect, preclinical model for RRP that will facilitate further study and treatment development for this intractable and devastating disease.

## 1. Introduction

Recurrent respiratory papillomatosis (RRP), largely caused by laryngeal infection with low-risk human papillomavirus (HPV) types 6 and 11, remains a rare but highly morbid human health concern without satisfactory treatment options [1]. RRP research has been slowed due to insufficient model systems [2]. In our companion paper [3], we described a novel mouse model of laryngeal papillomavirus infection using *Mus musculus* papillomavirus (MmuPV1). We found that, while vocal fold infections with MmuPV1 could cause benign disease, progression to cancer was frequent in our model, whereas it is rare in human RRP [4]. Viral gene expression is found in laryngeal tissues in our MmuPV1 infection model, as seen in human RRP [5,6,7]. However, we found MmuPV1 capsid production in squamous metaplasia only in our mouse model, while low-risk HPV capsid protein is found in sparse, superficial cells in human RRP lesions but is not absent [8]. Characterization of vocal fold epithelial changes induced by MmuPV1 is necessary to assess the utility of laryngeal MmuPV1 infection as a model for human RRP.

Vocal folds are paired, layered soft tissue structures within the larynx that contact each other and vibrate to produce voice. Vocal fold epithelium is critical to normal voice production [9] and withstands impact, tensile, and shear forces hundreds of times per second during conversational speech [10,11,12,13,14,15,16]. Like other tissue sites with high exposure to mechanical forces, vocal fold epithelium is stratified squamous [17]. Vocal folds are vulnerable to HPV6 and 11 infection and are the primary site of lesion growth in RRP [18]. 

RRP in humans induces changes in vocal fold epithelium at tissue and molecular levels. Hyperplasia in human RRP produces an irregular epithelial contour and mass lesions, which disrupt normal vocal fold vibration and voice production [19]. Healthy vocal fold epithelium is characterized by proliferating cells in the basal and parabasal layers and differentiated suprabasal cells [20,21,22]. Human RRP lesions are characterized by an expanded basal compartment, proliferating cells throughout epithelial layers, and decreased differentiation [23,24,25,26,27]. High-risk HPVs are known to suppress apoptosis in infected cells [28], and apoptosis may play a role in vocal fold epithelial diseases [29]. However, evidence for changes in apoptosis in human RRP lesions is equivocal [30,31,32,33]. Cell–cell junctions in vocal folds are primarily tight junctions; adherens junctions and gap junctions are also present [20,34]. RNA-seq experiments in RRP tissues have demonstrated decreased expression of cell junction and adhesion molecules [35]. Transmission electron microscopy (TEM) has demonstrated wide and irregular intercellular spaces in human RRP lesions compared with normal vocal fold epithelium [36,37,38]. Basement membrane between vocal fold epithelium and lamina propria contributes to barrier function of vocal fold mucosa [20,39]. High-risk HPV infection increases production of matrix metalloproteinases (MMPs), thinning the basement membrane, which is a necessary step in progression of high-risk HPV disease to invasive cancer [40]. This phenomenon is less clear in human RRP. TEM and histology studies have described vocal fold basement membrane in human RRP lesions as either discontinuous [36,37] or intact [38]. 

Murine laryngeal anatomy and physiology are grossly similar to those of humans but differ in several ways [41]. Mice and other rodents have a unique tissue fold called the ventral pouch, laryngeal pouch, or laryngeal sac in the ventral aspect of the larynx, superior to the vocal folds [41,42,43,44,45,46,47]. All evidence suggests that mouse vocalizations in the range of human hearing are produced using vocal fold vibration [48,49,50]. However, routine murine social communication is performed using ultrasonic vocalization [51], which is produced within the larynx, but not via vocal fold vibration [49,52,53]. The most recent evidence demonstrates that the ventral pouch is required for ultrasonic vocalization and suggests that vocal folds contribute by muscular action regulating ventral pouch size [52]. Like humans, murine vocal folds are layered, and their epithelium is stratified squamous [42,47,54]. Vocal fold epithelium is thinner in mice than in humans, comprising 2–4 cell layers rather than 5–10 [20,54]. Likely because of this difference, there is one layer of proliferating basal cells rather than two or three [54,55,56,57]. The same markers of basal cells (p63), proliferating cells (Ki67), differentiated cells (cytokeratin 13 (K13)), cell–cell junctions (E-cadherin, β-catenin, zonula occludens 1 (ZO-1)), and basement membrane (laminin, collagen IV) are present in the vocal fold epithelium of humans [20,21,22,34,58,59,60] and mice [34,47,54,55,56,57,61]. Mouse vocal fold epithelium is also positive for cytokeratin 8 (K8), a marker of simple and respiratory epithelium [54]. This is not seen in native adult human vocal folds [21,22,60]. Overall, however, vocal fold epithelium is similar between humans and mice. We hypothesize that laryngeal MmuPV1 infection recapitulates features of human RRP, specifically increased proliferation, decreased differentiation, altered cell–cell junctions, and thinning of the basement membrane.

Here, we characterize epithelial proliferation, apoptosis, differentiation, and barrier integrity in vocal fold disease induced by MmuPV1. We compared outcomes between control and diseased tissues at multiple stages of disease severity from mild dysplasia to invasive squamous cell carcinoma. We found that, consistent with hyperplasia in human RRP, the total number of epithelial cells increased in diseased vocal folds and increased with disease severity. Proliferating cells increased, while apoptotic cells did not change in diseased vocal folds. Like in human RRP, the basal compartment expanded, and differentiation markers decreased in diseased vocal fold epithelium. Cell–cell junctions decreased, as found in human RRP, but other interesting patterns of expression were noted, including nuclear expression of adherens junction marker E-cadherin and extramembranous, basal expression of tight junction marker ZO-1. Interestingly, laminin was found within diseased MmuPV1-infected vocal fold epithelium rather than being limited to the basement membrane zone. Overall, our findings demonstrate that laryngeal MmuPV1 infection is a useful, albeit imperfect, model of human RRP.

## 2. Materials and Methods

### 2.1. Animals, MmuPV1 Infection, Samples, and Pathology Grading

Adult male and female NOD scid gamma (NSG) mice [62] were used for this study. Animal procedures were approved by the University of Wisconsin-Madison Institutional Animal Care and Use Committee (IACUC #M005871) and conducted in accordance with the National Institutes of Health *Guide for the Care and Use of Laboratory Animals* [63]. Larynges were infected with MmuPV1 under endoscopy with and without vocal fold abrasion. Tissues were collected weeks 1, 2, 4, 8, and 12 post-infection and processed into formalin-fixed, paraffin-embedded (FFPE) slides. Hematoxylin and eosin (H&E)-stained slides were graded for disease severity in vocal folds, epiglottis and ventral pouch, arytenoids and aryepiglottic folds, proximal trachea, and hypopharynx by a Head and Neck Pathologist blinded to treatment group and timepoint post treatment. The disease was graded as mild, moderate, or severe dysplasia, or invasive cancer. Carcinoma in situ was graded as severe dysplasia. Detailed methods for animal experiments, MmuPV1 infection, sample collection, and pathology grading can be found in our companion paper [3].

### 2.2. Study Design

We compared quantitative and qualitative measures between mice with MmuPV1-induced dysplasia or cancer of the vocal folds, epiglottis/ventral pouch, arytenoids/aryepiglottic folds, proximal trachea, and hypopharynx, and equal numbers of uninfected controls. Samples represented a range of timepoints post infection within each degree of disease severity. Control animals were matched to diseased animals by injury status, sex, and timepoint post injury and/or infection. Control tissue sections were matched with diseased tissue sections in ventral-dorsal location within the larynx. Within diseased tissues, we also compared outcome measures among different degrees of disease severity. For proliferation assays, we stained and quantified sections from 6 mice per disease stage within each tissue, or the maximum number available if fewer than 6. For the apoptosis assay and markers of epithelial differentiation and barrier, we stained sections from 3 mice per disease stage within each tissue, or the maximum number available if fewer than 3. Sex, injury status, and timepoint were balanced within each disease stage for all experiments.

### 2.3. Immunofluorescence and Immunohistochemistry Staining

FFPE slides were stained following standard protocols for immunohistochemistry (IHC) and immunofluorescent (IF) staining. Briefly, sections were deparaffinized in xylene and rehydrated in a series of ethanols. For IHC, endogenous peroxidase was quenched in 3% hydrogen peroxide in methanol for 10 min. Antigen retrieval was performed by boiling slides in a microwave oven for 20 min in buffer. Antigen retrieval buffer was 10 mM citrate pH 6.0 for Ki67 or 1X Tris-EDTA pH 9.0 (Abcam ab93684, Cambridge, United Kingdom) for all other targets. Sections were permeabilized for 20 min in either 0.5% Triton X-100 for nuclear targets (p63, Ki67) or 0.2% PBS-Tween for non-nuclear targets (cytokeratins, ZO-1, E-cadherin, laminin). Slides were blocked for 1 h in 5% goat serum, then incubated in primary antibodies overnight at 4 °C. Primary and secondary antibodies are listed in Table 1. Select sections were incubated in 5% goat serum instead of primary antibody as a control for background staining. Slides were washed and incubated in secondary antibodies for 1 h at room temperature. For IHC, signal was detected with 3,3′-diaminobenzidine (DAB; SK-4100, Vector Laboratories, Inc., Burlingame, CA, USA) for 10 min, and then slides were counterstained for 1 min with hematoxylin QS (H-3404, Vector Laboratories, Inc.), dehydrated, cleared with xylene, mounted, and coverslipped. For IF, nuclei were counterstained with 1X Hoechst 33342 (H1399, Fisher Scientific, Hampton, NH, USA) for 10 min, and then slides were mounted and coverslipped with Prolong Diamond mounting media (P36970, Fisher Scientific), cured flat at room temperature in the dark for 24 h, and stored at 4 °C.

### 2.4. TUNEL Staining

Slides were stained for apoptotic cells using terminal deoxynucleotidyl transferase (TdT)-mediated deoxyuridine triphosphate (dUTP)-biotin nick end labeling (TUNEL) using the ApopTag Peroxidase In Situ Apoptosis Detection kit (S7100, MilliporeSigma, Burlington, MA, USA). Manufacturer protocols were modified to reduce background staining in mouse laryngeal and respiratory tissues. Briefly, slides were deparaffinized, rehydrated, and pretreated for 15 min with proteinase K (19133, Qiagen, Hilden, Germany) diluted to 20 ug/mL. Endogenous peroxidase was quenched in 0.3% hydrogen peroxide in PBS for 5 min. Sections were washed, incubated in equilibration buffer for 10 s, and then incubated in working strength TdT enzyme at 37 °C for 50 min. TdT enzyme was diluted to 3:100 with reaction buffer and diH2O (3 ul enzyme, 70 ul reaction buffer, and 27 uL diH2O per 100 uL). Select sections were incubated in diH2O instead of TdT enzyme as a negative control. The reaction was stopped by agitation followed by 10 min of incubation in working strength stop/wash buffer prepared per manufacturer protocol. Sections were then washed and incubated in anti-digoxigenin peroxidase for 30 min at room temperature. The signal was detected with DAB (SK-4100, Vector Laboratories, Inc.) for 10 s, and then slides were counterstained for 30 s with hematoxylin QS (H-3404, Vector Laboratories, Inc.), dehydrated, cleared with xylene, mounted, and coverslipped. To verify the kit activity with protocol modifications, as a positive control select sections were treated for 10 min with DNase I (EN0523, Fisher Scientific) diluted to 1000 U/mL in 1X buffer containing MgCl_2_ between proteinase K pretreatment and endogenous peroxidase quenching. 

### 2.5. MmuPV1 RNA in Situ Hybridization

In situ hybridization (ISH) for MmuPV1 E4 transcript was completed using RNAscope (2.5 HD Reagent Kit-Brown, 322300, Advanced Cell Diagnostics, Newark, CA, USA) with probes specific for MmuPV1 E4 (473281) according to the manufacturer’s instructions.

### 2.6. Image Acquisition

Images were acquired on a Nikon Eclipse Ti2 inverted microscope with NIS Elements software (Nikon, Tokyo, Japan). For quantitative proliferation and apoptosis assays, one 30× field was captured of each tissue of interest. This magnification captures a coronal section of the membranous mouse vocal fold. For other assays, areas of interest were photographed at 40×. Images were aligned to match tissue locations between diseased samples and injury-, sex-, and timepoint-matched controls. Images were adjusted in Adobe Photoshop (Adobe Inc., San Jose, CA, USA) or the GNU Image Manipulation Program (GIMP Development Team, volunteer group of software developers with no corporate headquarters) for consistency across slides relative to background fluorescence. For proliferation and apoptosis assays, total epithelial cells and DAB-labeled epithelial cells were quantified as described below. For IF assays, staining presence, relative intensity, and subcellular and tissue-level localization were described qualitatively.

### 2.7. Proliferation, Apoptosis, and Basal Cell Quantification

For proliferation and apoptosis assays, 30× images of Ki67- and TUNEL-stained tissues were opened in ImageJ (National Institutes of Health, Bethesda, MD, USA). Total epithelial cells and DAB-labeled cells were enumerated using an automated counting macro “BrdU Count v2.12.ijm” developed by Dr. David Ornelles (Wake Forest University, Winston-Salem, NC, USA) as previously described [64,65,66,67]. Prior to using the macro for these experiments, settings were adjusted for accuracy in laryngeal and respiratory epithelia in comparison to manual cell counts of 30× images of vocal fold and trachea (Figure S1). To quantify basal cells, total nuclei and p63-positive nuclei were counted in 40× images of p63- and Hoechst-stained epithelium using the ImageJ macro “RGB fluorescent cell count v1.32.ijm,” also developed by Dr. Ornelles.

### 2.8. Statistical Analysis

Labeled and unlabeled epithelial cells from the Ki67-stained slides were used for the analysis of total epithelial cell counts. Proliferation, apoptosis, and basal cell indices were calculated as percentages of Ki67+, TUNEL+, and p63+ cells, respectively. Data were tested for normality using the Kolmogorov–Smirnov test. Within each tissue type, difference between control and diseased tissues was tested with the paired T-test or Wilcoxon rank sum test, as appropriate for distribution. For Ki67 and TUNEL, differences among disease severity levels within a tissue and differences among laryngeal tissues (vocal folds, epiglottis, and arytenoids) were tested with the Kruskal–Wallis test with Dwass–Steel–Critchlow–Fligner (DSCF) pairwise post hoc test. For some analyses, data from laryngeal tissues were pooled. All samples stained for TUNEL were a subset of those stained for Ki67, and serial slides were stained. We analyzed the reliability of total epithelial cell counts between both stains using intraclass correlation coefficients (ICC) in a 2-way, mixed-effects, absolute agreement, single-rater model (called ICC(2,1) or ICC(A,1) by different authors [68,69,70]). ICCs were calculated in RStudio 1.4.1717 running R 3.4.3 (R Core Team, Vienna, Austria). All other statistical analyses were done with SAS Studio 3.8 running SAS 9.4.1 (SAS Institute, Inc., Cary, NC, USA) The alpha level for significance was 0.05.

## 3. Results

### 3.1. Proliferation

Epithelial cells were quantified for assays of proliferation (total number of epithelial cells and Ki67 index, n = 132 images at 30× magnification, 66 diseased and 66 uninfected controls) and apoptosis (TUNEL index, n = 84 images, 42 diseased and 42 controls). A total of 83 images were available from serial slides stained for both Ki67 and TUNEL. Total epithelial cell counts (DAB stained + only hematoxylin counterstained, control and diseased tissues) were assessed for intrarater reliability between the two assays. Overall, the reliability was excellent, particularly for the larynx and specifically the vocal folds (Table S1).

**Figure 1:**
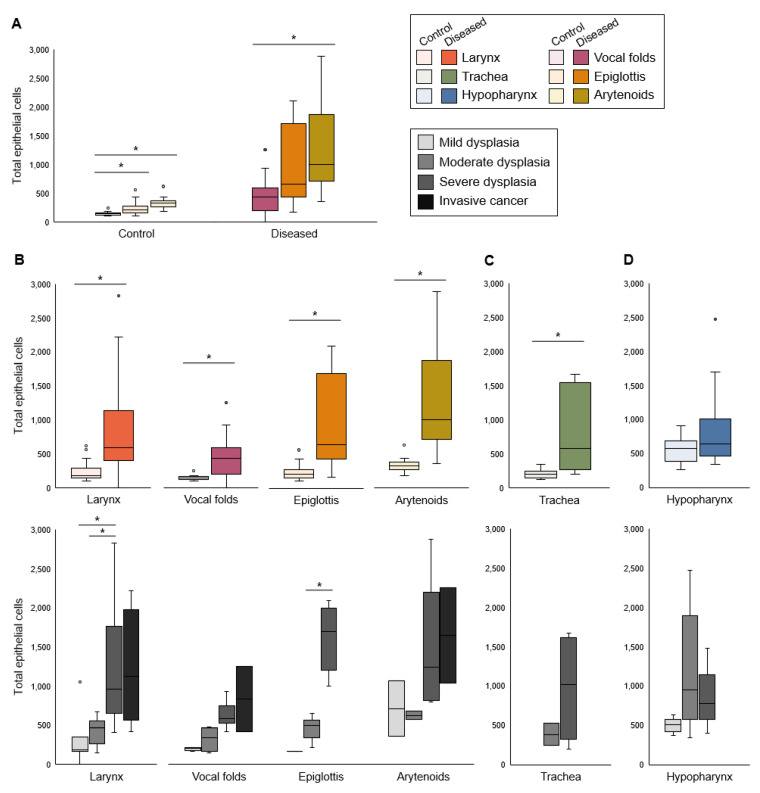
Total epithelial cells increased in diseased larynx and trachea. (**A**) Difference among laryngeal tissues in cell counts, n = 12–16 mice per tissue type in each condition. Kruskal–Wallis tests: control *p* < 0.0001, diseased *p* = 0.0034. *Significant post hoc DSCF pairwise tests: control vocal folds vs. epiglottis *p* = 0.0191, control vocal folds vs. arytenoids *p* < 0.0001, diseased vocal folds vs. arytenoids *p* = 0.0024. All other pairwise tests: *p* > 0.05. (**B**) Control vs. diseased laryngeal tissues and disease severity in laryngeal tissues. *Significant one-tailed Wilcoxon rank-sum tests for control vs. disease: larynx *p* < 0.0001, n = 41 mice per condition; vocal folds *p* < 0.0001, n = 16 mice per condition; epiglottis *p* = 0.0005, n = 13 mice per condition; arytenoids *p* < 0.0001, n = 12 mice per condition. Kruskal–Wallis tests for differences among severity levels: larynx *p* < 0.0001, n = 4–18 mice per disease stage; vocal folds *p* = 0.0240, n = 2–6 mice per disease stage; epiglottis *p* = 0.0079, n = 1–6 mice per disease stage; arytenoids *p* = 0.1979, n = 2–6 mice per disease stage. *Significant post hoc DSCF pairwise tests: mild vs. severe laryngeal dysplasia *p* = 0.0073, moderate vs. severe laryngeal dysplasia *p* = 0.0006, moderate vs. severe epiglottic dysplasia *p* = 0.0110. All other pairwise tests: *p* > 0.05. (**C**) Control vs. diseased trachea and disease severity in trachea. *Significant one-tailed Wilcoxon rank-sum test for control vs. disease: *p* = 0.0050, n = 8 mice per condition. Kruskal–Wallis test for differences among severity levels: *p* = 0.3173, n = 2–6 mice per disease stage. (**D**) Control vs. diseased hypopharynx and disease severity in hypopharynx. One-tailed Wilcoxon rank-sum test for control vs. disease: *p* = 0.1012, n = 17 mice per condition. Kruskal–Wallis test for differences among severity levels: *p* = 0.0862, n = 5–6 mice per disease stage.

Cell counts differed among laryngeal tissues (Figure 1A). The number of epithelial cells was increased in all diseased laryngeal tissues compared to controls and increased with disease severity (Figure 1B). Cell count also increased in diseased proximal trachea but did not differ by severity (Figure 1C). In the hypopharynx, the median number of epithelial cells did not differ between diseased tissue and uninfected controls (Figure 1D).

**Figure 2:**
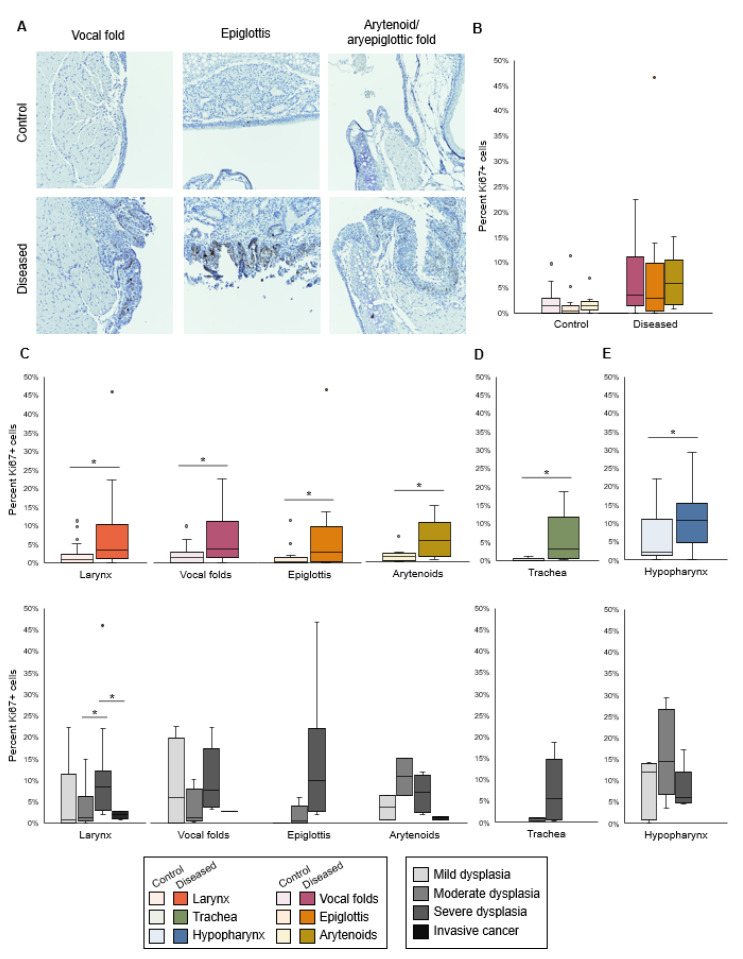
Proliferation index increased in diseased larynx, trachea, and hypopharynx. (**A**) Ki67 IHC stain in control and diseased vocal folds, epiglottis, and arytenoids, 30× magnification. (**B**) Difference among laryngeal tissues in % Ki67+ cells, n = 12-16 mice per tissue type in each condition. Kruskal–Wallis tests: control *p* = 0.1647, diseased *p* = 0.6574. (**C**) Control vs. diseased laryngeal tissues and disease severity in laryngeal tissues. *Significant one-tailed Wilcoxon rank-sum tests for control vs. disease: larynx *p* < 0.0001, n = 41 mice per condition; vocal folds *p* = 0.0122, n = 16 mice per condition; epiglottis *p* = 0.0218, n = 13 mice per condition; arytenoids *p* = 0.0104, n = 12 mice per condition. Kruskal–Wallis tests for differences among severity levels: larynx *p* = 0.0212, n = 4-18 mice per disease stage; vocal folds *p* = 0.3098, n = 2–6 mice per disease stage; epiglottis *p* = 0.0392, n = 1–6 mice per disease stage; arytenoids *p* = 0.1375, n = 2–6 mice per disease stage. *Significant post hoc DSCF pairwise tests: moderate vs. severe laryngeal dysplasia *p* = 0.0482, severe laryngeal dysplasia vs. invasive cancer *p* = 0.0464. All other pairwise tests: *p* > 0.05. (**D**) Control vs. diseased trachea and disease severity in trachea. *Significant one-tailed Wilcoxon rank-sum test for control vs. disease: *p* = 0.0052, n = 8 mice per condition. Kruskal–Wallis test for differences among severity levels: *p* = 0.3173, n = 2–6 mice per disease stage. (**E**) Control vs. diseased hypopharynx and disease severity in hypopharynx. *Significant one-tailed Wilcoxon rank-sum test for control vs. disease: *p* = 0.0137, n = 17 mice per condition. Kruskal–Wallis test for differences among severity levels: *p* = 0.3645, n = 5–6 mice per disease stage.

In uninfected larynges, Ki67 was expressed in rare basal epithelial cells (Figure 2A). Ki67+ cells were found above the basal layer in diseased laryngeal epithelium (Figure 2A). The proliferation index did not differ by subsite within the larynx (Figure 2B). Quantitatively, there were more proliferating epithelial cells in infected larynges than in uninfected larynges (median 3.4%, IQR 1.3–10.0%, vs. median 0.9%, IQR 0.0–2.2%, *p* < 0.0001, Figure 2C). The relationship between Ki67 and disease severity was unclear. Group difference among disease severity levels was significant in the larynx as a whole when considering pooled data and appeared to be driven by a very high proliferation index in one epiglottis (Figure 2C). Differences by severity were not significant in vocal folds or arytenoids. Proliferation index increased in diseased proximal trachea and hypopharynx but did not differ by disease severity (Figure 2D–E). Overall, total epithelial cell counts and Ki67 staining demonstrated quantifiable hyperplasia and proliferation in MmuPV1-induced laryngeal disease.

### 3.2. Apoptosis

**Figure 3:**
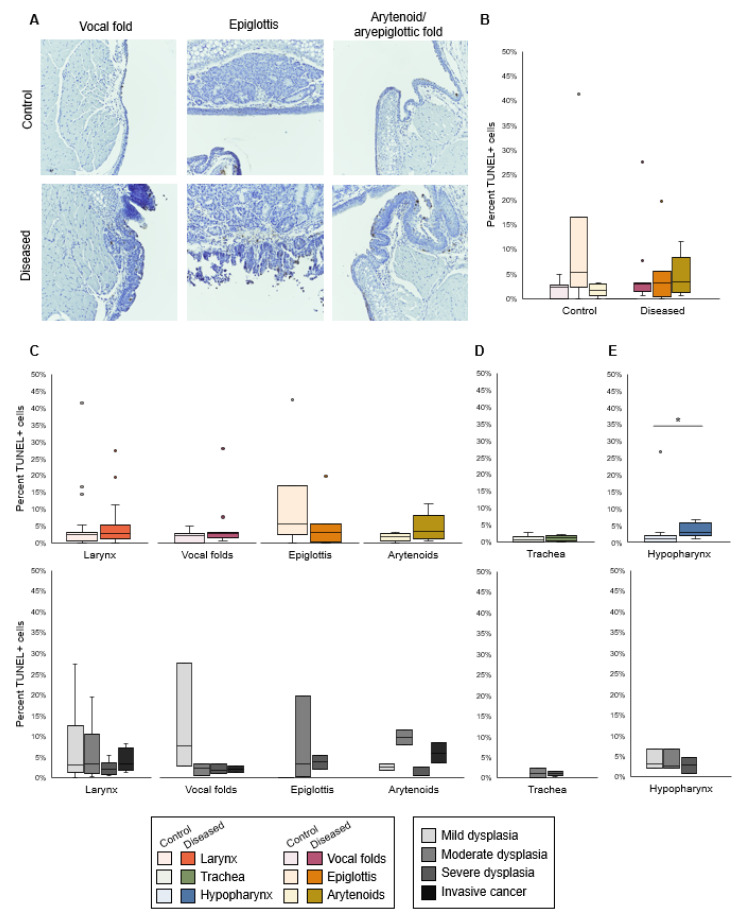
Apoptosis index did not differ in diseased larynx or trachea but increased in diseased hypopharynx. (**A**) TUNEL stain in control and diseased vocal folds, epiglottis, and arytenoids, 30× magnification. (**B**) Difference among laryngeal tissues in % TUNEL+ cells, n = 7–11 mice per tissue type in each condition. Kruskal–Wallis tests: control *p* = 0.0690, diseased *p* = 0.8296. (**C**) Control vs. diseased laryngeal tissues and disease severity in laryngeal tissues. Two-tailed Wilcoxon rank-sum tests for control vs. disease: larynx *p* = 0.1794, n = 27 mice per condition; vocal folds *p* = 0.2493, n = 11 mice per condition; epiglottis *p* = 0.4423, n = 7 mice per condition; arytenoids *p* = 0.0634, n = 9 mice per condition. Kruskal–Wallis tests for differences among severity levels: larynx *p* = 0.6510, n = 4–9 mice per disease stage; vocal folds *p* = 0.3347, n = 2–3 mice per disease stage; epiglottis *p* = 0.3189, n = 1–3 mice per disease stage; arytenoids *p* = 0.0846, n = 2–3 mice per disease stage. (**D**) Control vs. diseased trachea and disease severity in trachea. Two-tailed Wilcoxon rank-sum test for control vs. disease: *p* = 0.4680, n = 6 mice per condition. Kruskal–Wallis test for differences among severity levels: *p* = 1.0000, n = 2–4 mice per disease stage. (**E**) Control vs. diseased hypopharynx and disease severity in hypopharynx. *Significant two-tailed Wilcoxon rank-sum test for control vs. disease: *p* = 0.0374, n = 9 mice per condition. Kruskal–Wallis test for differences among severity levels: *p* = 0.8752, n = 3 mice per disease stage.

TUNEL+ cells were found primarily in apical epithelium (Figure 3A). The apoptosis index was low but highly variable in both uninfected (median 2.4%, IQR 0.5–3.2%) and diseased (median 2.9%, IQR 1.4–5.4%) laryngeal tissues. The number of TUNEL+ cells did not differ by subsite within the larynx (Figure 3B). Apoptosis index did not differ between diseased and control larynges or trachea but increased in diseased hypopharynx (Figure 3C–E). Disease severity was not associated with apoptosis in any tissue.

### 3.3. Epithelial Differentiation

**Figure 4:**
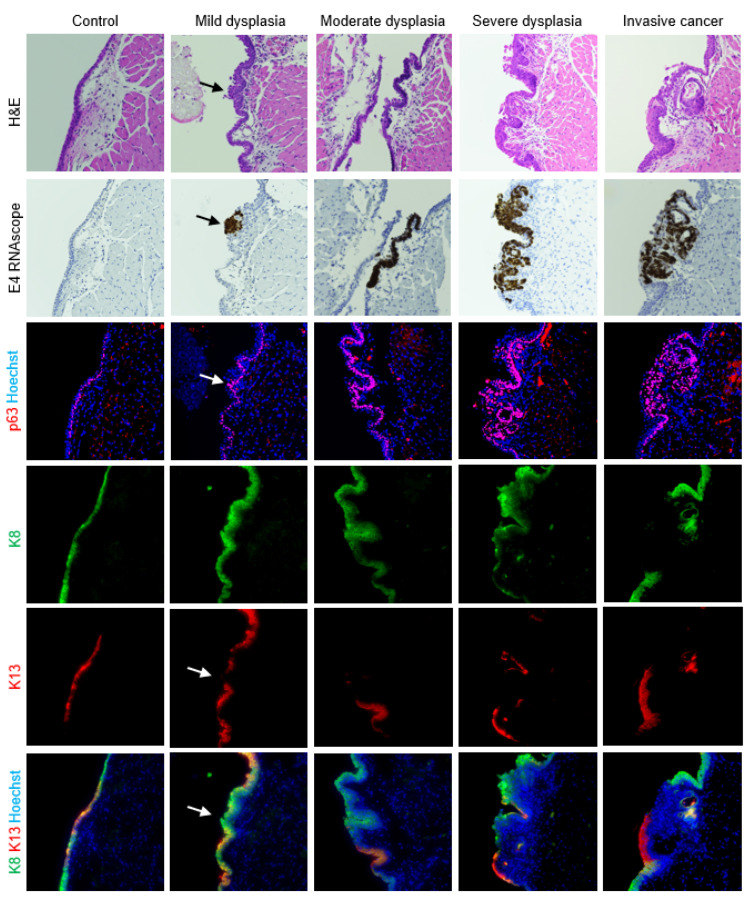
Expanded epithelial basal compartment in MmuPV1-induced vocal fold disease. Serial sections of control and diseased vocal folds stained with H&E, MmuPV1 E4 RNAscope ISH, costained p63 (red) and Hoechst (blue) IF, and costained K8 (green), K13 (red), and Hoechst (blue) IF shown both as separate channels and merged. 40× magnification. Arrows: MmuPV1-positive focal dysplasia with suprabasal p63 and reduced K13. Some H&E and RNAscope images reproduced with permission from our companion paper [3].

**Figure 5:**
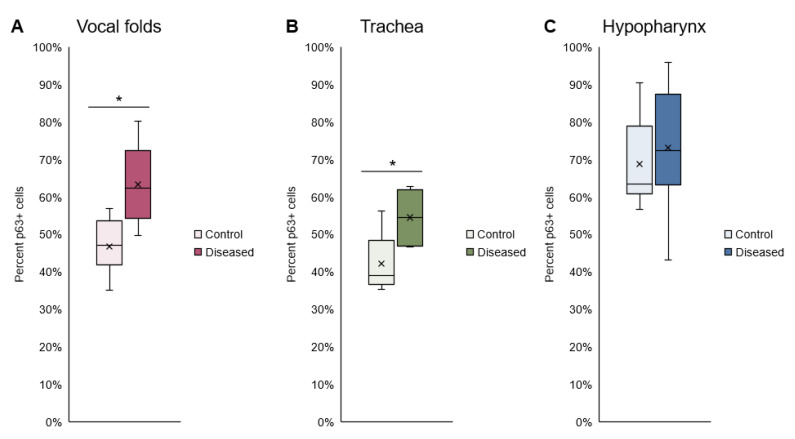
Basal cells increased in diseased vocal folds and trachea after MmuPV1 infection. Percent p63-positive cells in control vs. diseased tissues. *Significant paired T-test. x labels group means. (**A**) Vocal folds: *p* = 0.0003, n = 10–12 mice per group. (**B**) Trachea: *p* = 0.0161, n = 6 mice per group. (**C**) Hypopharynx: *p* = 0.5592, n = 8 mice per group.

As expected, normal uninfected vocal fold epithelium was characterized by p63 expression in basal cells, K13 in apical cells, and K8 throughout the epithelium (Figure 4). In mild vocal fold dysplasia induced by MmuPV1 infection, hyperplastic foci with decreased K13 and suprabasal p63 were found. Cell layers increased in hyperplastic epithelium, and K8 gradually increased from basal to apical layers. These changes were more pronounced in moderate dysplasia. In severe vocal fold dysplasia, there was an expansion of the basal compartment. p63 was found in all epithelial layers as well as lining epithelial invaginations. K13 was severely reduced, as was K8 to a lesser extent, and the expression of both was limited to apical cells. Squamous cell carcinomas in MmuPV1-infected vocal folds were characterized by loss of both K13 and K8 in areas of the epithelium. p63-positive epithelial cells increased from 46.7% ± 7.2% in normal vocal folds to 63.3% ± 10.1% in diseased vocal folds (*p* = 0.0003, Figure 5A). In the epiglottis/ventral pouch and arytenoid epithelium, the expression of p63, K13, and K8 in mirrored findings in vocal folds except for some suprabasal p63 expression in arytenoids and variable K13 expression in epiglottis at baseline (Figure S2). 

**Figure 6:**
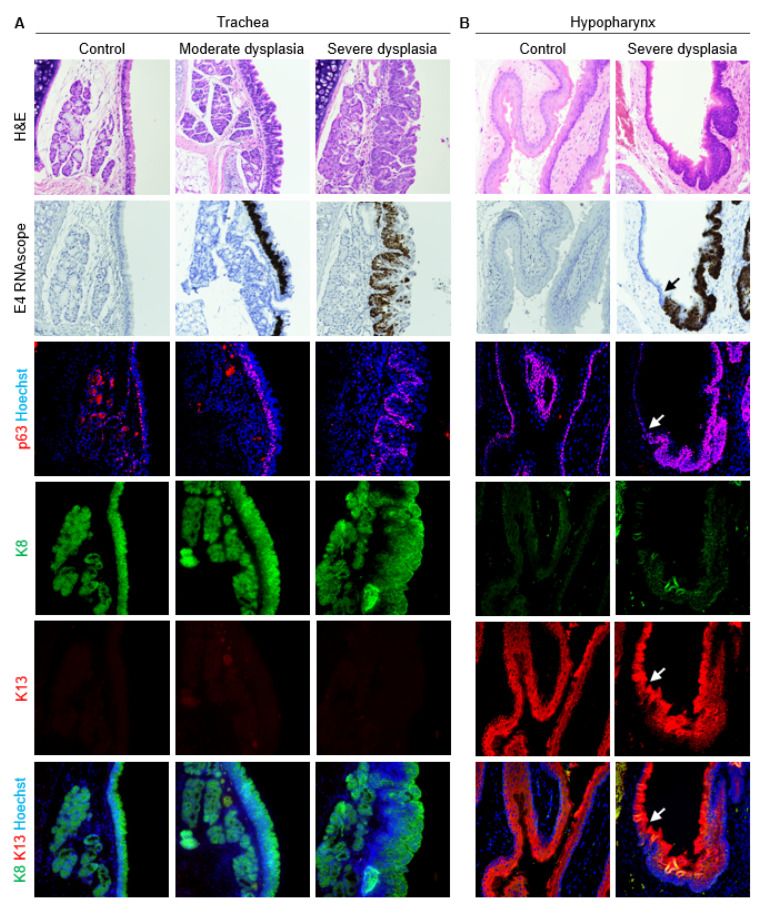
Expanded epithelial basal compartment in MmuPV1-induced disease in proximal trachea and hypopharynx. Serial sections of control and diseased tissues stained with H&E, MmuPV1 E4 RNAscope ISH, costained p63 (red) and Hoechst (blue) IF, and costained K8 (green), K13 (red), and Hoechst (blue) IF shown both as separate channels and merged. 40× magnification. Some H&E and RNAscope images reproduced with permission from our companion paper [3]. (**A**) Trachea. (**B**) Hypopharynx. Arrows indicate transition between normal, nondiseased epithelium and dysplastic, MmuPV1-positive epithelium coinciding with expanded basal compartment.

Respiratory epithelium of the proximal trachea was K8+ and K13- with and without MmuPV1-induced disease (Figure 6A). Like laryngeal epithelium, dysplasias were characterized by an expansion of the basal compartment with p63 positivity and weak K8 staining. p63-positive basal cells increased from 42.1 ± 7.8% of epithelial cells to 54.5 ± 7.0% in diseased trachea (*p* = 0.0161, Figure 5B). Uninfected hypopharynx was characterized by a p63+ basal layer and K13+ cells above the basal layer, with weak to absent K8 expression (Figure 6B). In MmuPV1-induced dysplasias, the basal compartment expanded, characterized by p63 positivity and reduced K13. Percent p63-positive basal cells did not significantly increase in diseased hypopharynx (68.7 ± 11.8% vs. 73.1 ± 16.8%, *p* = 0.5592, Figure 5C). Thus, in respiratory, intermediate, and nonkeratinized stratified squamous epithelia inside and outside the larynx, MmuPV1-induced disease was characterized by an increase in undifferentiated cells.

### 3.4. Epithelial Barrier

**Figure 7:**
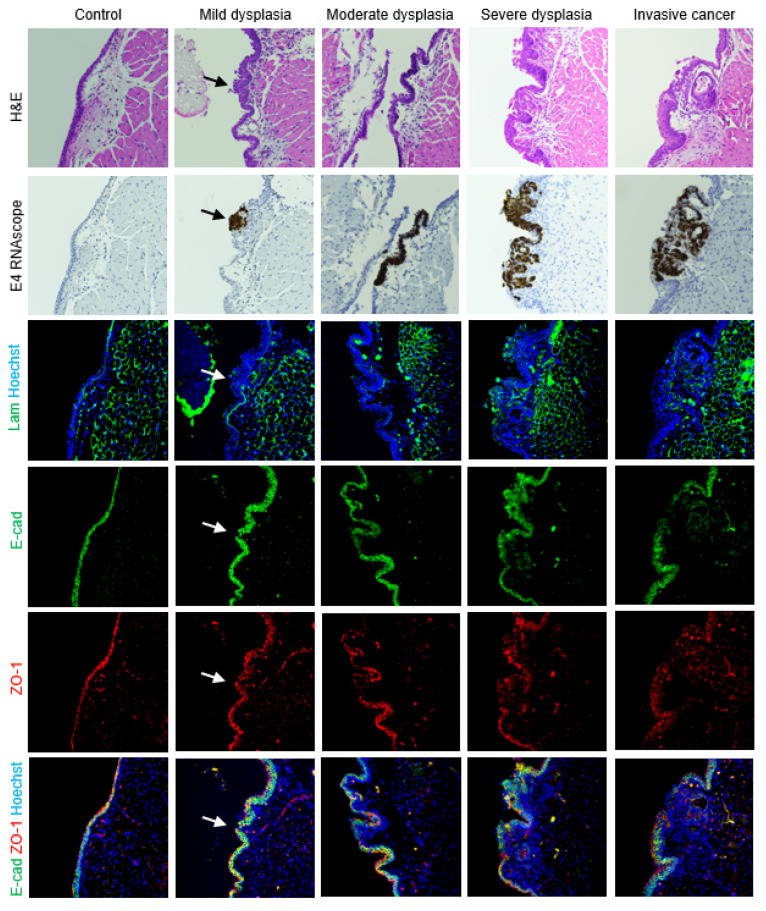
Perturbed epithelial barrier in vocal folds with disease induced by MmuPV1. Serial sections of control and diseased vocal folds stained with H&E, MmuPV1 E4 RNAscope ISH, costained laminin (green) and Hoechst (blue) IF, and costained E-cadherin (green), ZO-1 (red), and Hoechst (blue) IF shown both as separate channels and merged. 40× magnification. Arrows: MmuPV1-positive focal dysplasia with epithelial laminin and reduced E-cadherin and ZO-1. Some H&E and RNAscope images reproduced with permission from our companion paper [3].

**Figure 8:**
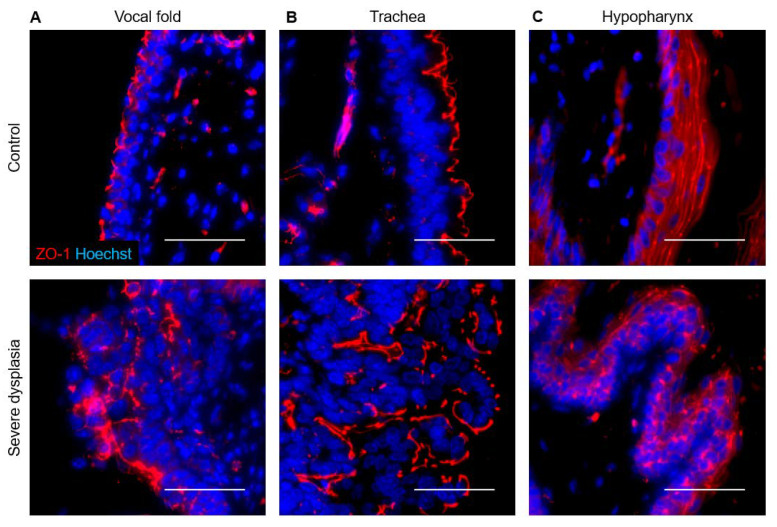
Disorganized ZO-1 in MmuPV1-induced severe dysplasias. Control and diseased tissues costained for ZO-1 (red) and Hoechst (blue) IF. Top row: 40× magnification. Bottom row: higher magnification of areas indicated by boxes. Scale bars = 50 um. (**A**) Vocal fold. (**B**) Trachea. (**C**) Hypopharynx.

In uninfected vocal folds, laminin labeled the basement membrane below the epithelium (Figure 7). The adherens junction marker E-cadherin was expressed in cell membranes throughout epithelium, and the tight junction marker ZO-1 was expressed in cell membranes in more apical cells (Figure 8A). Barrier proteins were slightly disorganized in mild vocal fold dysplasia, with focally decreased E-cadherin and ZO-1 and laminin expression in epithelial cells (Figure 7). E-cadherin was decreased in moderate dysplasia, and a ZO-1 signal appeared in the basal layer around folds of hyperplastic epithelium, with a strong signal that was not limited to cell membranes. These changes were also observed in severe dysplasia (Figure 8A), as was laminin expression throughout the epithelium. In vocal fold cancer induced by MmuPV1, epithelial laminin expression persisted, and cell junction proteins became patchy and lost in areas (Figure 7). Barrier protein expression in control and diseased epithelia of the epiglottis and arytenoids wase similar to vocal folds (Figure S3). 

**Figure 9:**
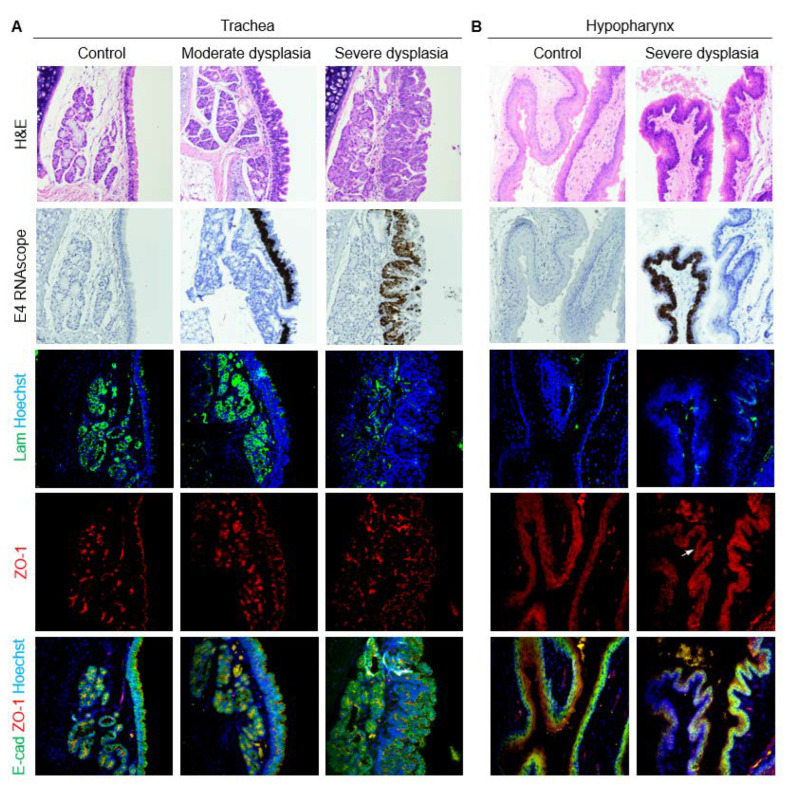
Perturbed epithelial barrier in trachea and hypopharynx with disease induced by MmuPV1. Serial sections of control and diseased tissues stained with H&E, MmuPV1 E4 RNAscope ISH, costained laminin (green) and Hoechst (blue) IF, and costained E-cadherin (green), ZO-1 (red), and Hoechst (blue) IF shown both in red channel and merged. 40× magnification. Some H&E and RNAscope images reproduced with permission from our companion paper [3]. (**A**) Trachea. (**B**) Hypopharynx. Arrow: basal ZO-1 beyond cell membranes in MmuPV1-positive dysplastic epithelium.

**Figure 10:**
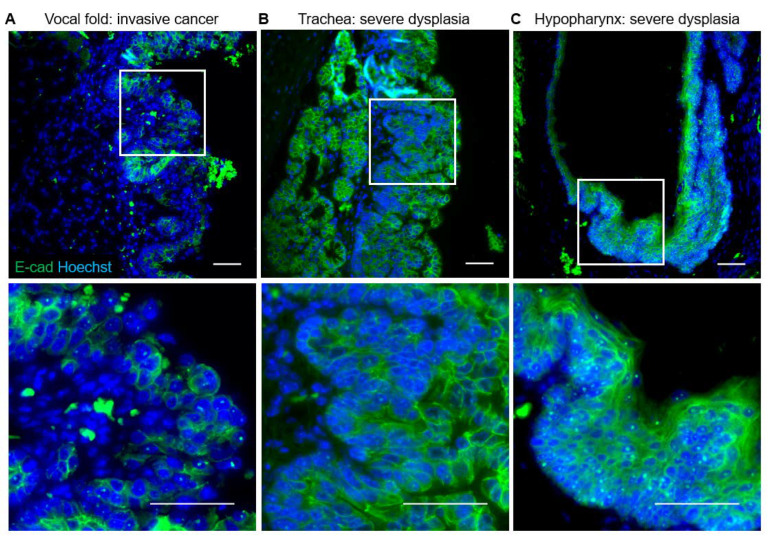
Nuclear E-cadherin in MmuPV1-induced cancers and severe dysplasias. Diseased tissues costained for E-cadherin (green) and Hoechst (blue) IF. Top row: 40× magnification. Bottom row: higher magnification of areas indicated by boxes. Scale bars = 50 um. (**A**) Vocal fold. (**B**) Trachea. (**C**) Hypopharynx.

In the uninfected trachea, laminin labeled the basement membrane with some staining of cilia, E-cadherin labeled cell membranes throughout the epithelium, and ZO-1 formed a distinct line along the luminal surface of cells (Figure 8B, Figure 9A). In dysplasias, E-cadherin expression decreased in the expanding basal compartment. ZO-1 increased to form two distinct lines in moderate dysplasia and surrounded folds of hyperplastic epithelium in severe dysplasia (Figure 8B, Figure 9A). Changes in laminin with MmuPV1-induced dysplasia mirrored ZO-1, with laminin appearing to support layers of hyperplastic cells (Figure 9A). In control hypopharyngeal epithelium, laminin marked the basement membrane, and cell junction proteins were membranous, with E-cadherin expression stronger closer to basal layers and ZO-1 expression stronger in suprabasal and apical layers (Figure 8C, Figure 9B). In severe dysplasia, laminin was occasionally found in the epithelium. ZO-1 expression appeared in basal layers and expanded beyond cell membranes in severe dysplasia (Figure 8C, Figure 9B). E-cadherin expression was decreased (Figure 9B). Nuclear E-cadherin was observed in severe dysplasias and cancers of all tissues, most notably the hypopharynx (Figure 10). Overall, MmuPV1-associated disease was characterized by disorganized and mislocalized barrier proteins, with ZO-1 and laminin apparently supporting increased layers and folds of hyperplastic epithelium.

## 4. Discussion

We compared quantitative and qualitative measures of proliferation, apoptosis, epithelial differentiation, and epithelial barrier between mice with MmuPV1-induced disease of the larynx, proximal trachea, and hypopharynx and equal numbers of uninfected controls. Our findings support our hypothesis that laryngeal MmuPV1 infection increase proliferation and disrupt differentiation and barriers, characteristic features of human RRP. Similarities and differences between NSG laryngeal epithelial disease associated with MmuPV1 and human RRP are summarized in Table 2.

Consistent with obvious hyperplasia on histology, the total number of epithelial cells increased in laryngeal tissues with MmuPV1-induced disease. In uninfected laryngeal tissues, the proliferation index (% Ki67 + cells) was about 1%. This is consistent with the literature reporting very few proliferating cells in adult mouse vocal folds [55,57,73]. Proliferation index increased to a median of 3.4% in diseased murine laryngeal tissues, and Ki67-expressing cells were found above the basal layer of the epithelium. A similar expansion of proliferating cells has been observed in RRP lesions [23] and in MmuPV1-induced lesions of the oral cavity and the anogenital tract [74,75,76,77]. Like the larynx, we found that proliferation and hyperplasia increased in trachea with MmuPV1-induced disease. In the diseased hypopharynx, however, proliferation increased without resulting in quantifiable hyperplasia. Murine hypopharynx has more epithelial cell layers than the larynx at baseline (5–7 vs. 2–4 in our observations), so perhaps the difference induced by disease in hypopharynx was not great enough to be statistically significant. 

There was no effect of MmuPV1-induced disease on apoptosis in laryngeal epithelium. High-risk HPV-infected cells resist apoptosis [28]. Studies of apoptosis in RRP tissues compared with normal laryngeal tissues have found both pro- and anti-apoptotic effects on upstream effectors and inhibitors of apoptosis [30,31,32,33]. A common downstream effect of both intrinsic and extrinsic apoptosis pathways is DNA fragmentation, which is detected by TUNEL. One study found no difference in DNA fragmentation between RRP tissues and normal laryngeal tissues [32]. The relationship between apoptosis in both RRP disease and MmuPV1 laryngeal infection remains unclear based on the literature and our findings. In contrast to the larynx, apoptosis increased in hypopharynx with MmuPV1-induced disease. MmuPV1 has been shown to increase Bak, an effector of apoptosis, in the skin of medically immunosuppressed mice [78]. Unlike the larynx, the stratified squamous epithelium of mouse hypopharynx is keratinized [79]. It is possible that the hypopharynx epithelium more closely resembles skin in the effect of MmuPV1 on apoptotic pathways.

As expected, we observed an expansion of the basal compartment of epithelial cells in tissues with MmuPV1-induced disease. These cells were characterized by p63-positivity and the absence of differentiation markers specific to each tissue. Percent basal cells as a fraction of epithelial cells increased in diseased larynx and trachea. In the larynx, both K13 and K8 marked epithelium above the basal layer as previously shown [54]. K13- cells increased in dysplasias, and K8- cells increased in severe dysplasias. A patchy loss of both K13 and K8 was observed in apical layers of laryngeal cancers induced by MmuPV1. Similarly, in tracheal dysplasia, the p63+/K8- compartment was expanded. In hypopharynx, p63+/K13- cells above the basal layer visibly increased in severe dysplasia. However, like the difference in proliferating Ki67+ cells, the difference in basal p63+ cells was not statistically significant in quantitative analysis. Decreased K13 and other markers of epithelial differentiation have been reported in RRP [24,25]. In skin, MmuPV1 increases the basal cell compartment of infected epithelium and decreases cytokeratin 10 (K10)-positive differentiated cells [77]. Thus, our results are consistent with the literature on epithelial differentiation in both MmuPV1-induced disease and human RRP.

In MmuPV1-induced laryngeal disease, tight junction marker ZO-1 localized to basal cells of invaginating, hyperplastic epithelium, as opposed to primarily apical cells in normal epithelium. Its expression extended into the cytoplasm, beyond the clear line of membranous positivity seen in uninfected cells. In tracheal disease, ZO-1 remained membranous but was expressed as multiple discrete layers that increased with hyperplasia and disease severity. RNA-seq experiments have shown downregulated epithelial barrier components in both high- and low-risk HPV infection, including RRP tissues [35,80]. E6 proteins of high-risk HPVs disrupt tight junctions through direct interaction with ZO-1 [81,82] and other cellular proteins containing a post synaptic density protein (PSD95)/Drosophila disc large tumor suppressor (DLG)/ZO-1 (PDZ) domain [83]. However, E6 proteins of low-risk HPV types 6 and 11 lack a PDZ-binding motif [83]. ZO-1 has not been specifically studied in RRP. To our knowledge, the effect of MmuPV1 on cell junctions has not been previously explored. MmuPV1 E6 contains a putative PDZ-binding motif, but not in the extreme carboxy-terminus like high-risk HPV E6 proteins [84]. A straightforward decrease in ZO-1 may not be expected in either RRP or in MmuPV1-induced disease. 

We found that membranous E-cadherin expression decreased and became patchy in MmuPV1-induced disease of the larynx. RRP clinical data for E-cadherin are not available for comparison. In cervical cancer, E-cadherin protein level is reduced and is inversely correlated with high-risk HPV E6 and E7 levels [85]. In genital lesions caused by low-risk HPV6 and 11, the same types that cause RRP, E-cadherin staining is generally reduced, but the level of reduction is heterogeneous across patients [86]. In addition to a reduction in membranous expression, nuclear E-cadherin expression was found in severe laryngeal dysplasias and cancers induced by MmuPV1. This has been observed in a number of cancer types and has been validated by subcellular fractionation, but requires an antibody against the cytoplasmic domain rather than the extracellular domain of E-cadherin to be revealed upon immunostaining [87,88,89,90,91,92]. Nuclear E-cadherin may regulate transcription of many signaling pathways involved in tumorigenesis [87,89,91].

We hypothesized that MmuPV1 would decrease laminin in laryngeal disease. Laminin has not been examined in MmuPV1-associated diseases of other tissues. The basement membrane is disrupted in some TEM studies of RRP lesions [36,37]. The invasion of epithelial cells through basement membrane is a hallmark of invasive cancer. High-risk HPVs increase many MMPs [40], including types that are known to degrade laminin and other basement membrane components [93,94]. We found that laminin expression was disorganized in MmuPV1-induced laryngeal disease but that, contrary to expectations, expression increased in diseased epithelium. Laminins are heterotrimers containing α, β, and γ chains. The primary laminin detected by our antibody is laminin-111 (formerly called laminin-1), i.e., α1/β1/γ1 [95,96]. Laminin-111 and laminin-332 (formerly laminin-5) have been reported in the vocal fold basement membrane of humans and rodents [61,97,98,99]. Many cancers, including laryngeal squamous cell carcinoma, express laminin-111 and -332 throughout the tumor tissue [100,101,102]. Both laminins contain peptides that promote tumor growth [100,101]. In our model, laminin expression in MmuPV1-induced laryngeal disease was consistent with its expression in laryngeal cancer. 

As summarized in Table 2, the laryngeal epithelial response to MmuPV1-induced disease grossly resembled findings in RRP, despite the subtle differences discussed above. The major limitation of the present work is the exclusive use of immunocompromised mice. There are important interactions between the immune system and epithelial cells that may be clinically relevant in RRP patients, but that our experiments could not address. For example, cytotoxic CD8+ T cells are decreased in RRP lesions [103,104], and stress keratin K17 transcripts are upregulated [105]. Transcriptomic analysis has revealed molecular subtypes of RRP associated with clinical course. Patients with more aggressive disease had lesions characterized by increased low-risk HPV gene expression, reduced expression of genes involved in interferon signaling, antigen presentation, and T cell function, fewer CD4+ and CD8+ T cells in lesions, and increased expression of certain cytokeratin genes, including K17 [7]. A recent study using MmuPV1 found a mechanistic link between these phenomena in skin, demonstrating that MmuPV1 increased K17 in lesions, which prevented CD8+ T cell infiltration via disrupted chemokine signaling and allowed lesion growth [64]. As another example, E-cadherin binds Langerhans cells to epithelial cells and is required for Langerhans cell maturation [106,107]. High- and low-risk HPV infections decrease both E-cadherin and Langerhans cells in cervical mucosa [86]. RRP lesions have immature Langerhans cells with a defective response to cytokines [108,109]. Since immunodeficiency in NSG mice includes a lack of T cells and defective Langerhans cells at baseline, another mouse strain is required to understand interactions among keratins, cell adhesion molecules, and the immune response to MmuPV1 in the larynx and to generate a model that parallels human immune response to low-risk HPVs in RRP. Immunocompromised mouse strains are highly vulnerable to MmuPV1, but certain immunocompetent strains also develop infection and disease in various tissues [84,110,111]. This warrants investigation in the larynx. An immunocompetent animal model of laryngeal papillomavirus infection could be used to reveal larynx-specific mechanisms of viral immune evasion. For example, the interactions discussed above among keratins, cell junction proteins, and innate and adaptive immune cells in papillomavirus infection could have implications for RRP. However, they have not been mechanistically studied in the larynx, which has a unique immunologic milieu [112]. With mechanisms of viral immune evasion in the larynx defined, this model could then be used to discover methods to overcome immune evasion and elicit host immune-mediated resolution of RRP.

## 5. Conclusions

MmuPV1 increased proliferation in infected laryngeal tissues, expanded the basal compartment of cells, and decreased differentiated cells. There was no effect of MmuPV1 on apoptosis. Tight junction marker ZO-1 was mislocalized rather than decreased. These findings are consistent with findings in RRP lesions and low-risk HPV biology. The effects of MmuPV1-induced disease on adherens junction marker E-cadherin and basement membrane marker laminin resembled human cancers, but the status of these biomarkers in RRP has not been reported. We conclude that MmuPV1 infection of the mouse larynx provides a useful, if imperfect, preclinical model for RRP.

## Figures and Tables

**Table 1:** Primary and secondary antibodies.

Primary Antibody	Species	Dilution	Supplier	Catalog #	Application
Ki67	Rabbit	1:50	Abcam	ab16667	IHC
Cytokeratin 8 (K8)	Rabbit	1:200	LS Bio, Seattle, WA USA	LS-B7928	IF
Cytokeratin 13 (K13)	Mouse	1:100	Fisher Scientific	MA1-35542	IF
p63	Mouse	1:200	Biocare Medical, Pacheco, CA, USA	CM163A	IF
Zonula occludens 1 (ZO-1)	Mouse	1:200	Fisher Scientific	33-9100	IF
E-cadherin (E-cad)	Rabbit	1:200	Cell Signaling Technology, Danvers, MA. USA	3195S	IF
Laminin (Lam)	Rabbit	1:200	Abcam	ab11575	IF
**Secondary antibody**	**Species**	**Dilution**	**Supplier**	**Catalog #**	**Application**
Horseradish peroxidase (HRP)	Goat anti-rabbit	1:500	Jackson Immuno Research Labs, West Grove, PA, USA	111-035-144	IHC
Alexa Fluor 488	Goat anti-rabbit	1:500	Fisher Scientific	A-11008	IF
Cy3	Goat anti-mouse	1:500	Jackson Immuno Research Labs	115-166-003	IF

**Table 2:** Epithelial similarities and differences between vocal fold disease in NSG mice infected with MmuPV1 and RRP.

Disease Feature	Mouse Vocal Fold + MmuPV1	Human RRP
Hyperplasia	Yes	Yes [19,71,72]
Increased proliferation	Yes	Yes [23]
Decreased differentiation	Yes	Yes [24,25]
Decreased apoptosis	No	Unclear [30,31,32,33]
Altered barrier	Yes	Yes [35,36,37]

## Data Availability

All relevant data are provided within the article and supplementary materials.

## Supplementary Materials

The following supporting information can be downloaded at: https://www.mdpi.com/article/10.3390/v14051059/s1, Table S1: Intrarater reliability of total epithelial cell counts; Table S2: Correlations between proliferation and apoptosis indices; Figure S1: Cell counting results did not differ between manual and automated counting methods; Figure S2: Epithelial differentiation markers in epiglottis and arytenoids with disease induced by MmuPV1; Figure S3: Epithelial barrier markers in epiglottis and arytenoids with disease induced by MmuPV1.
