# Supplementary material for: Expanded Basal Compartment and Disrupted Barrier in Vocal Fold Epithelium Infected with Mouse Papillomavirus MmuPV1
Source: Viruses. 2022 May 16;14(5):1059. doi: 10.3390/v14051059 (PMC9146965; doi:10.3390/v14051059)
Supplement: Supplementary file 1 [file viruses-14-01059-s001.zip › viruses-1693905-supplementary.pdf]

SUPPLEMENTARY MATERIALS

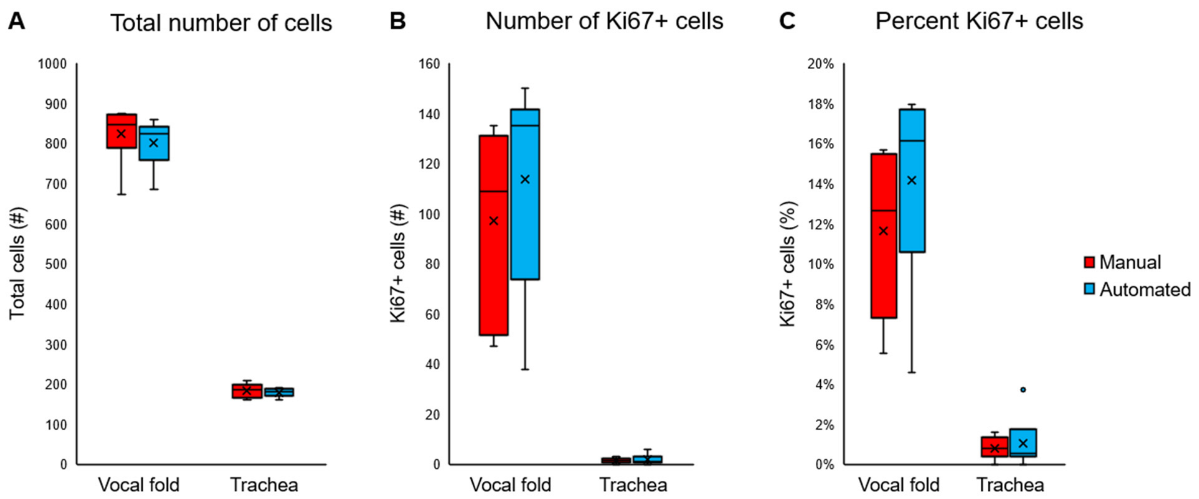

Figure S1. Cell counting results did not differ between manual and automated counting methods. Serial slides (n = 6) from one mouse with airway dysplasia were stained for Ki67 and counterstained with hematoxylin. Vocal fold and trachea were imaged at 30X magnification. Total epithelial cells (Ki67+ and Ki67-) and Ki67+ epithelial cells were quantified in ImageJ by both manual cell counting and an automated counting macro. All distributions were normal (Kolmogorov–Smirnov test, all $p > .05$), and there was no evidence for unequal variances between methods (Levene's test, all $p > .05$). Results of paired T-tests: **(a)** Total number of epithelial cells in manual vs. automated counting: vocal folds p = .0832, trachea p = .5800. **(b)** Number of Ki67+ epithelial cells in manual vs. automated counting: vocal folds p = .1104, trachea p = .5761. **(c)** Percent of Ki67+ epithelial cells in manual vs. automated counting: vocal folds p = .1037, trachea p = .3632.

SUPPLEMENTARY MATERIALS

Table S1. Intrarater reliability of total epithelial cell counts.

Tissue	n	ICC	95% CI	*p*	Reliability[348]
All tissues	83	0.91	0.87-0.94	<.0001*	Excellent
Larynx	53	0.93	0.90-0.96	<.0001*	Excellent
Vocal folds	22	0.98	0.96-0.99	<.0001*	Excellent
Epiglottis	13	0.91	0.78-0.96	<.0001*	Excellent
Arytenoids	18	0.92	0.83-0.96	<.0001*	Excellent
Trachea	12	0.98	0.95-0.99	<.0001*	Excellent
Hypopharynx	18	0.79	0.58-0.90	<.0001*	Good

Total epithelial cells were quantified in 30X images from 2 serial sections. Data were pooled as follows: Vocal folds + Epiglottis + Arytenoids = Larynx. Larynx + Trachea + Hypopharynx = All tissues. Intrarater reliability was assessed using a 2-way mixed effects, absolute agreement, single rater model[348] using the psych(ICC) function in R. ICC2 in psych(ICC) = ICC(2,1)[349] = ICC(A,1).[350,353] CI: confidence interval. *$p < .05$

SUPPLEMENTARY MATERIALS

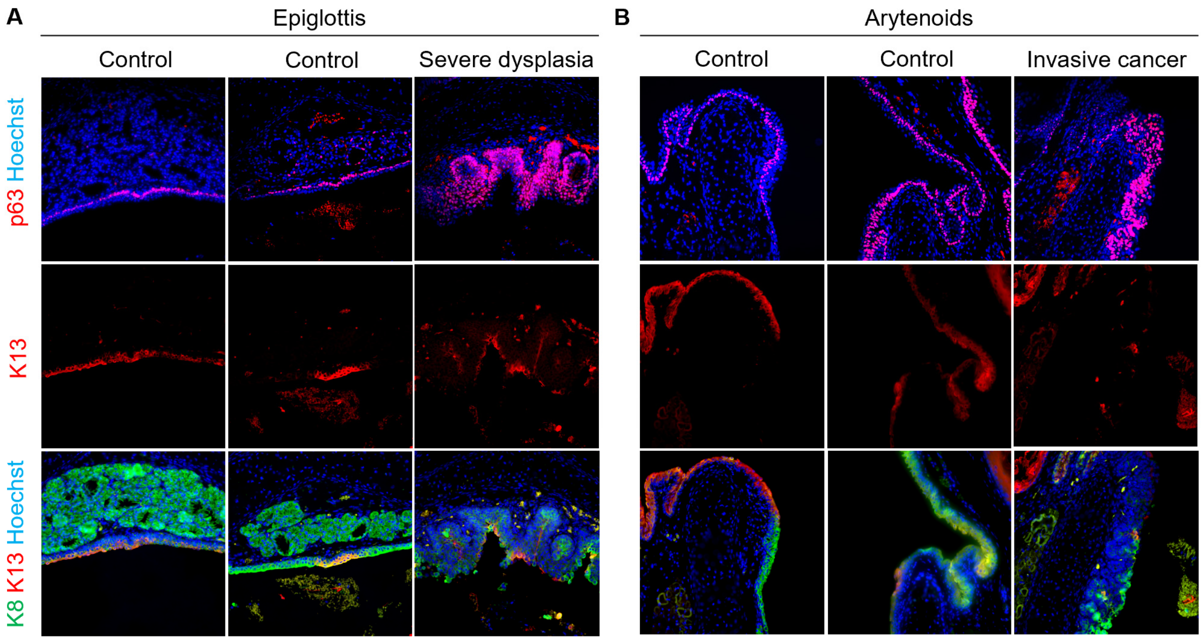

Figure S2. Epithelial differentiation markers in epiglottis and arytenoids with disease induced by MmuPV1. Serial sections of control and diseased tissues stained with p63/Hoechst merged IF, K13 IF, and K8/K13/Hoechst merged IF. 40X magnification. **(a)** Epiglottis. Two control samples are shown to depict variability in K13 in uninfected epiglottic epithelium. **(b)** Arytenoids. Two control samples are shown to depict some p63 above the basal layer of epithelium and variability in K13 expression depending on anterior-posterior location within the larynx in uninfected arytenoids.

SUPPLEMENTARY MATERIALS

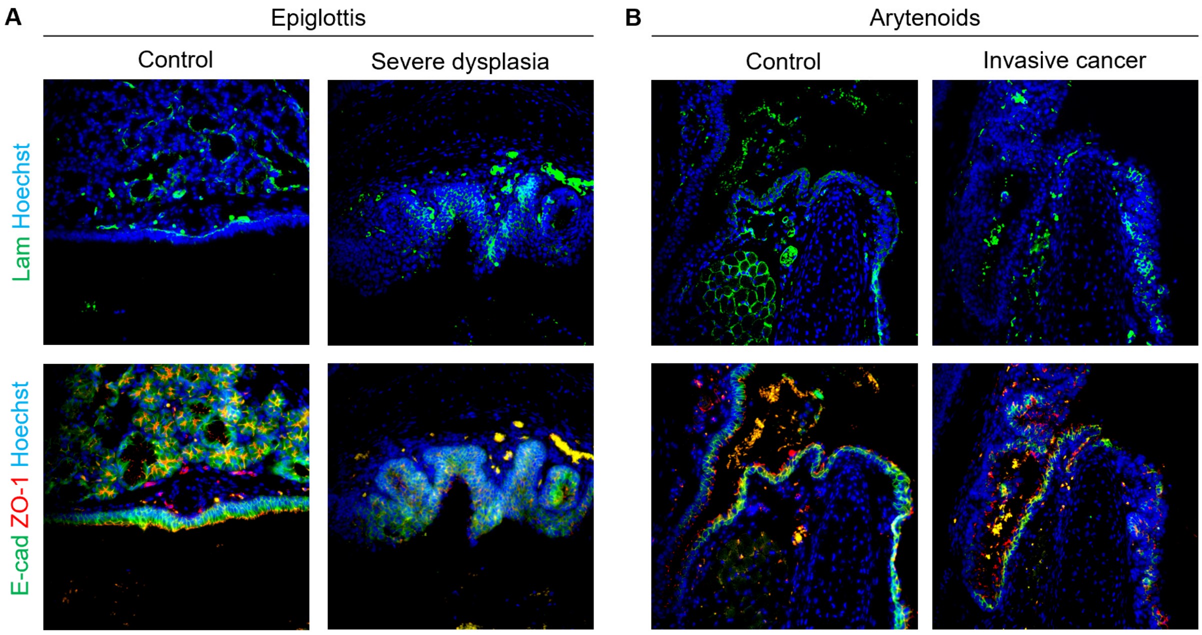

Figure S3. Epithelial barrier markers in epiglottis and arytenoids with disease induced by MmuPV1. Serial sections of control and diseased tissues stained with laminin/Hoechst merged IF and E-cadherin/ZO-1/Hoechst merged IF. 40X magnification. **(a)** Epiglottis. **(b)** Arytenoids.
